# Critical Pathways for Transforming the Energy Future: A Review of Innovations and Challenges in Spent Lithium Battery Recycling Technologies

**DOI:** 10.3390/ma18132987

**Published:** 2025-06-24

**Authors:** Zhiyong Lu, Liangmin Ning, Xiangnan Zhu, Hao Yu

**Affiliations:** 1 College of Energy and Mining Engineering, Shandong University of Science and Technology, Qingdao 266590, China; lzy5762@163.com; 2 College of Energy Storage Technology, Shandong University of Science and Technology, Qingdao 266590, China; ninglm90@sdust.edu.cn; 3 Jiangsu Osume Technology Co., Ltd., Nantong 226000, China

**Keywords:** retired lithium-ion batteries, advanced recycling technology, green leaching, material regeneration, resource sustainability

## Abstract

In the wake of global energy transition and the “dual-carbon” goal, the rapid growth of electric vehicles has posed challenges for large-scale lithium-ion battery decommissioning. Retired batteries exhibit dual attributes of strategic resources (cobalt/lithium concentrations several times higher than natural ores) and environmental risks (heavy metal pollution, electrolyte toxicity). This paper systematically reviews pyrometallurgical and hydrometallurgical recovery technologies, identifying bottlenecks: high energy/lithium loss in pyrometallurgy, and corrosion/cost/solvent regeneration issues in hydrometallurgy. To address these, an integrated recycling process is proposed: low-temperature physical separation (liquid nitrogen embrittlement grinding + froth flotation) for cathode–anode separation, mild roasting to convert lithium into water-soluble compounds for efficient metal oxide separation, stepwise alkaline precipitation for high-purity lithium salts, and co-precipitation synthesis of spherical hydroxide precursors followed by segmented sintering to regenerate LiNi_1/3_Co_1/3_Mn_1/3_O_2_ cathodes with morphology/electrochemical performance comparable to virgin materials. This low-temperature, precision-controlled methodology effectively addresses the energy-intensive, pollutive, and inefficient limitations inherent in conventional recycling processes. By offering an engineered solution for sustainable large-scale recycling and high-value regeneration of spent ternary lithium ion batteries (LIBs), this approach proves pivotal in advancing circular economy development within the renewable energy sector.

## 1. Introduction

Under the synergistic influence of the Paris Agreement’s climate governance regime and national “dual-carbon” strategies, the global electrification momentum in automotive industries has already materialized [1], as evidenced by sustained growth in electric vehicle (EV) sales markets [2]. Current global market projections indicate annual EV sales will surpass 15 million units by 2025 [3], while cumulative deployments are expected to reach 63 million units in 2025 and further escalate to 175 million units by 2030 according to International Energy Agency forecasts [4]. This market expansion not only underscores the irreversible trajectory of transportation electrification but also portends impending systemic challenges in lifecycle management for EVs’ core energy storage components [5].

Lithium-ion batteries, as the mainstream energy storage devices in the EV sector, have achieved industry-wide acceptance owing to their technical feasibility and proven commercial viability [6,7]. Market projections indicate these batteries will maintain their technological dominance through the next decade, owing to their superior energy density [8], demonstrated safety profiles [9], and efficient cycling performance [10]. Lithium-ion battery demand is experiencing exponential growth, with forecasts predicting 222 GWh requirements by 2025 and nearly 1000 GWh by 2030 [4]. However, despite this promising outlook, the irreversible lithium dendrite formation on electrode surfaces during prolonged use gradually degrades capacity, limiting operational lifespans to approximately 5–6 years [11,12,13].

The concurrent surge in the EV adoption and inherent battery lifespan constraints will precipitate massive spent battery volumes [14], with annual spent lithium-ion batteries projected to reach 400,000–500,000 tons across all chemistries by 2025 [15]. This figure will escalate to 3 million tons annually by 2030 [16], accumulating to over 11 million tons of total spent battery stockpiles [17]. The resulting spent battery influx creates an urgent imperative to develop sustainable disposal solutions [18].

The environmental risks and resource potential of spent lithium-ion batteries are closely interconnected. While these waste materials pose environmental hazards due to volatile organic compounds and toxic heavy metal components [19], they simultaneously represent valuable secondary resources. Spent LIBs contain concentrated reserves of strategically important metals—particularly cobalt and lithium with concentrations significantly exceeding those found in natural ores. Recycling these materials offers substantial economic benefits while demonstrating measurable reductions in carbon emissions compared to primary metal extraction processes.

Decommissioned lithium batteries pose significant contamination risks, encompassing bioaccumulation effects of heavy metals, persistent organic electrolyte pollution, and potential transboundary fluoride migration [20]. Flammability and explosivity hazards have also been identified in end-of-life lithium-ion cells [21]. The environmental threat is directly linked to their composition: Sadeghi reports that lithium-ion batteries contain approximately 5% toxic electrolytes and over 26% hazardous heavy metals by weight [22]. Quantitative analysis by Lin reveals that each 4000-ton batch of spent batteries contains 1100 tons of heavy metals and 200 tons of toxic electrolytes [23]. These constituents represent primary environmental and health hazards [24]. Heavy metals like copper demonstrate bioaccumulation through food chains, causing chronic toxicity in organisms including humans [25]. Electrolyte components including dimethyl carbonate, methyl ethyl carbonate, and LiPF_6_ exhibit high volatility, toxicity, and flammability [26,27]. Their aqueous decomposition generates hydrofluoric acid (HF), contributing to fluorine pollution and ozone depletion [25]. Electrolyte hydrolysis and combustion further release water-polluting compounds like formaldehyde, methanol, and acetaldehyde [4]. Beyond these primary pollutants, auxiliary components exacerbate environmental risks: binder materials release HF under thermal stress, while separator combustion generates CO, aldehydes, and organic pollutants [28,29]. These pollutants demonstrate synergistic toxicity, creating composite contamination effects with ecological impacts far exceeding simple additive effects, thereby posing systemic threats to regional environmental security.

The environmental risks of discarded lithium-ion batteries coincide with their valuable metal resources. Spent LIBs contain substantial quantities of strategic metals, with concentrations significantly exceeding those found in primary mineral resources [30,31]. For example, typical retired lithium-ion batteries contain 5–20% cobalt (Co) [32], a concentration that is 5000 to 20,000 times higher than that in natural ores [8]. These batteries also contain 5–7% lithium (Li), surpassing both lithium brine (0.01–0.02%) and ore deposits (0.5–2%) [32]. Nickel (Ni) concentrations typically range between 5 and 10% [33]. Utilizing recovered materials in battery production demonstrates a 52% reduction in carbon emissions compared to virgin resource processing [34].

These dual attributes position spent LIBs as both environmental pollutants and valuable secondary resources, necessitating high-value, low-impact recycling solutions. Market forecasts indicate imminent industry growth, with global recycling market values projected to reach 23.72 billion by 2030 [32]. Liu’s independent projection aligns closely, estimating a $22.8 billion market valuation by 2030 [35].

The technological advancement within lithium-ion battery recycling systems directly determines resource recovery efficiency and environmental risk mitigation capabilities. A comprehensive overview of these technologies reveals that advancing technological solutions represents a critical pathway for addressing industrial challenges while simultaneously accelerating the transition toward sustainable circular economy models. This study systematically examines lithium-ion battery architectures, pre-treatment protocols, pyrometallurgical processes, and hydrometallurgical methodologies, offering detailed technical evaluations and comparative analyses of each approach. Additionally, a systematic comparison was conducted on the extraction methodologies for valuable metal elements in pyrometallurgical and hydrometallurgical processes during the recovery of metals from spent LIBs. Finally, building on these technological foundations, we propose an integrated synergistic recycling framework leveraging differential material property utilizations. The process employs low-temperature embrittlement flotation for efficient cathode/anode material separation, overcoming traditional physical separation limitations that require high-purity feedstock while reducing organic binder processing energy demands. An innovative low-temperature molten salt roasting system addresses thermal energy barriers inherent in conventional high-temperature operations, enabling lithium-transition metal separation at significantly reduced temperatures with minimized pollutant generation. Through sequential leaching, impurity removal, and dynamic ionic concentration regulation, the process resolves elemental recovery and compositional homogeneity challenges in complex systems. Finally, segmented sintering enables direct conversion of waste electrode materials into high-purity anode precursors, reducing reagent consumption and emissions. This integrated methodology signifies a paradigm shift in the “low-energy green regeneration” of ternary battery systems, providing critical insights for the development of circular economy frameworks within the new energy sector.

## 2. The Structure of Lithium-Ion Batteries

Lithium-ion batteries exhibit remarkable design versatility, with form factors tailored to meet specific energy storage requirements across diverse applications. Common configurations include cylindrical, prismatic, button, and pouch cells [9]. Smartphones typically employ pouch cells, whose thin and lightweight form factors align with device compactness requirements, while electric vehicles predominantly utilize cylindrical (e.g., Tesla Model 3/Y 21,700 cells) and prismatic (e.g., BYD Blade battery) geometries optimized for energy density, structural integrity, and thermal management [36]. The electric vehicle sector currently constitutes the largest market for LIBs, driving continuous technological innovation.

**Figure 1:**
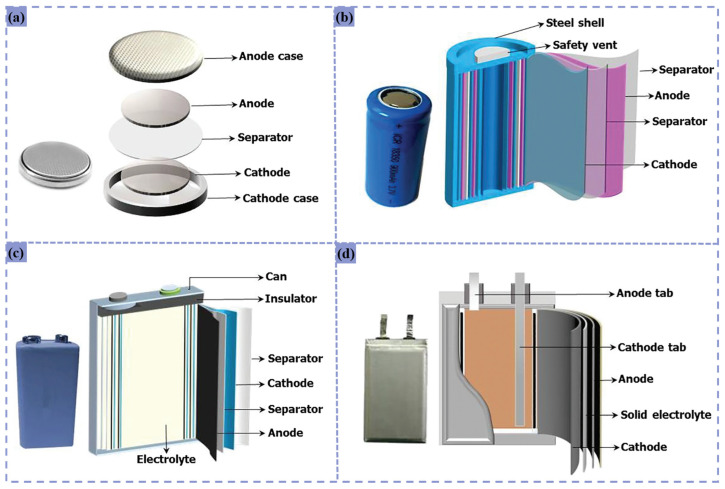
Schematic illustration of typical rechargeable battery configurations: (**a**) coin, (**b**) cylindrical, (**c**) prismatic, and (**d**) pouch shapes [38].

Vehicle-grade LIBs incorporate layered components including anodes, separators, and cathodes arranged in either spiral-wound configurations (cylindrical cells) or multi-layer stacked sheet architectures (prismatic/pouch cells), all housed within robust steel enclosures. Electrolyte infusion and hermetic sealing are achieved through precision welding processes, as illustrated in Figure 1 [34]. This structural design serves dual purposes: safeguarding sensitive internal components while enhancing mechanical robustness and operational safety. Key internal components comprise cathode materials, anode materials, electrolytes, separators, and bipolar current collectors (aluminum foil for cathodes, copper foil for anodes) [37]. These elements operate synergistically to enable efficient electrochemical energy storage and discharge functionalities.

Cathode materials in lithium-ion batteries contain strategic elements including Ni, Co, Mn, Li, and Al, comprising active metal oxides, aluminum foil substrates, and polyvinylidene fluoride (PVDF) binders [28]. Chemically, these oxides encompass lithium cobalt oxide (LCO, LiCoO_2_), lithium manganese oxide (LMO, LiMn_2_O_4_), lithium iron phosphate (LFP, LiFePO_4_), nickel–cobalt–aluminum oxide (NCA, Li_x_NiCoAlO_2_), and nickel–manganese–cobalt oxide (NMC, LiNiCoMnO_2_) formulations [39]. PVDF binders mechanically anchor these oxides to aluminum current collectors, forming structurally stable electrodes [40]. Notably, cathode materials represent the primary focus of LIB recycling efforts due to their strategic element composition [41].

Anode structures typically feature carbonaceous materials (graphite/graphene) dispersed with polymeric binders, uniformly coated onto copper foil current collectors [33]. This configuration enhances both electrochemical conductivity and mechanical electrode stability. The electrolyte system consists of solvent media and lithium salts, with lithium hexafluorophosphate (LiPF_6_) being the most prevalent electrolyte salt [37]. This formulation ensures efficient ionic conductivity while maintaining electrochemical stability during battery operation, thereby guaranteeing optimal performance and service life.

## 3. The Pre-Treatment Processes in Lithium-Ion Battery Recycling

Pre-treatment represents an essential preliminary step in the recycling of decommissioned lithium-ion batteries. This critical phase encompasses operations including electrical discharge, mechanical disassembly, comminution, and material segregation. These procedures serve dual purposes: neutralizing residual electrical energy to ensure operational safety and liberating embedded metal components to facilitate subsequent material recovery. By decoupling interconnected battery components, pre-treatment optimizes the efficiency of downstream separation processes, thereby establishing foundational conditions for effective resource recovery.

Pre-treatment of spent lithium-ion batteries mandates thorough discharge to mitigate risks of short-circuiting or thermal runaway during subsequent processing. Discharge protocols typically target terminal voltages below 0.5 V, with sodium chloride (NaCl) solutions representing the most common discharge medium [42]. Key parameters influencing discharge efficiency include electrolyte concentration, ambient temperature, and immersion duration. Studies demonstrate that 5% NaCl solutions require 48 h immersion periods for adequate discharge, as evidenced by Jiang and Han’s protocols [43,44]. Zhou observed that 10% NaCl solutions achieve the 0.5 V threshold within 10 min, suggesting concentration-dependent efficiency gains [45]. Nshizirungu’s work with LiCoO_2_ cells further supports high-concentration applications, employing 20% NaCl solutions for 48 h immersions to ensure complete discharge [46].

Temperature management also critically impacts discharge performance. Mondal’s investigations into LiFePO_4_ batteries revealed that elevated temperatures (35 °C) reduce residual energy by approximately 50% compared to 25 °C conditions, with only marginal improvements at 45 °C [3]. This phenomenon arises from accelerated lithium-ion diffusion kinetics and reduced internal resistance at moderate temperatures. However, operating above 35 °C introduces thermal runaway risks without significant efficiency benefits, establishing 35 °C as the optimal discharge environment [3].

Laboratory-scale disassembly of lithium-ion batteries typically begins with manual separation of the cathode foil to isolate the active material from the current collector [33]. This process often involves controlled disassembly within inert atmospheres to mitigate safety risks, with Yang demonstrating argon-filled glove box protocols for fully discharged NCM battery cells [47]. Manual disassembly enables precise component separation and remains the predominant laboratory method for isolating internal battery materials. Jiang exemplified this approach by using pliers to separate discharged LIB components from their metallic casings, facilitating material recovery [43]. Post-disassembly, electrode strips are frequently cleansed with dimethyl carbonate (DMC) to eliminate residual electrolyte contaminants [44].

Alternative mechanical separation techniques have also been reported. Yuan described a multi-stage process involving initial shear crushing of discharged LIBs, followed by magnetic/air classification to remove metallic casings and plastic films. Subsequent pyrolysis at 600 °C for 30 min induces material magnetization, enabling magnetic separation of cathode plates (5000 G field strength) from non-magnetic anode components [48].

Following disassembly, cathode active material must be separated from the current collector substrate to enable subsequent metallurgical recovery of strategic metals. Conventional approaches employ mechanical comminution followed by particle size classification. Jiang demonstrated this method by processing cathode plates in a shear crusher (FS-100, China) for 30 s, followed by sieving through a 200-mesh aperture to isolate particles ≤0.074 mm, which are enriched in active materials [43].

Advanced separation techniques have also been developed. Wang employed non-thermal plasma (NTP) treatment under 300 W oxygen atmospheres for 2000 s to selectively degrade PVDF binders through bond scission [49]. Subsequent high-speed pulverization (10,000 rpm, 100 s) and multi-stage sieving (16-mesh to 60-mesh screens) enabled precise particle fractionation. Particles >0.25 mm were identified as aluminum foil fragments, while the <0.25 mm fraction contained cathode material powders with 95.69% separation efficiency and only 0.02 wt% aluminum contamination. This approach demonstrates significant advantages in achieving high-purity material streams for downstream processing [49].

**Figure 2:**
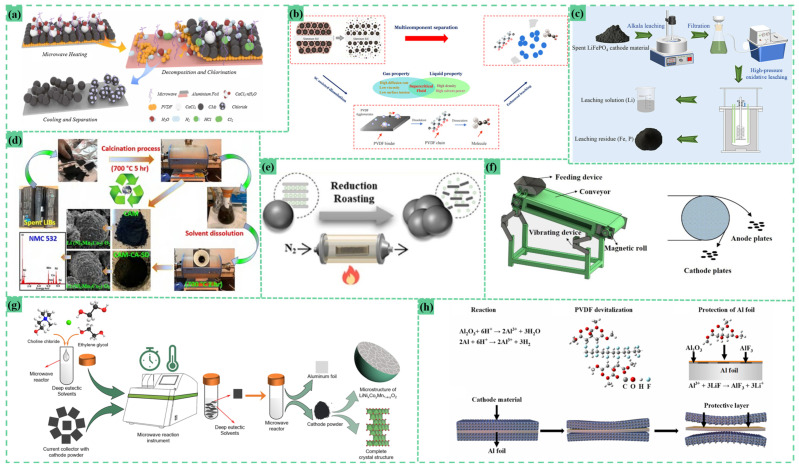
(**a**) Schematic diagram of the whole recycling process by microwave roasting [13]; (**b**) flow chart of SC-CO_2_ extraction of cathode mater [42]; (**c**) metal recovery process of spent LiFePO_4_ cathode material under high-pressure oxidation acid leaching [50]; (**d**) spent lithium-ion battery recycling and cathode material processing flow diagram [33]; (**e**) reduction roasting pre-treatment for spent lithium-ion batteries [51]; (**f**) magnetic separation method [48]; (**g**) schematic diagram of an efficient separation of cathode powder from the Al foil by using microwave-assisted DES [40]; (**h**) mechanism and schematic of Al foil and cathode material separation [44].

Conventional approaches relying on mechanical crushing and sieving exhibit inherent limitations, particularly incomplete aluminum foil dissociation and active material embedment issues. Over-grinding induces particle agglomeration, causing cathode materials to become trapped within aluminum substrate pores or encapsulated by foil fragments, ultimately compromising recovery yields. To mitigate these challenges, innovative delamination techniques (Figure 2) are gaining traction, offering enhanced separation efficiency between aluminum substrates and cathode active materials while maintaining material integrity for subsequent recovery processes.

The ultrasonic water-impact method represents a versatile and cost-effective pre-treatment approach applicable to various cathode material systems, demonstrating significant potential for industrial-scale deployment. Zhou’s investigations revealed that water introduction during cathode material comminution effectively suppresses dust generation from active materials and conductive agents while providing thermal stabilization through heat absorption during fragmentation processes [45]. This aqueous optimization strategy has been successfully implemented in multiple studies: Yang employed ultrasonic water bath treatment to achieve ternary cathode (NCM) powder delamination from cathode substrates, followed by thermal purification at 650 °C to decompose PVDF binders and conductive carbon additives [47]. Yuan et al. adopted a multi-stage aqueous mechanical processing protocol, initially applying water-assisted impact crushing under 400 r/min agitation for 60 min to dislodge fine electrode particles from aluminum current collectors [48]. Subsequent sieving through 0.045 mm apertures enabled separation of aluminum foil from lithium-bearing electrode materials, though partial dissolution of lithium salts (Li_2_CO_3_, Li_2_O) necessitated pH adjustment to 7.0 to mitigate aluminum corrosion during this aqueous processing stage [48].

The organic solvent stripping method enables efficient active material–aluminum foil separation through solvent-specific interactions, offering advantages in environmental compatibility, material preservation, and solvent recovery. Tong demonstrated this approach by immersing NCM523 cathode fragments in N-methyl-2-pyrrolidone (NMP) at 70 °C for 40 min, achieving complete aluminum substrate detachment [52]. Han optimized a methanol–citric acid (MeOH-CA) solvent system containing 30 wt% CA and 3 wt% H_2_O at 45 °C, achieving >99.5% separation efficiency with minimal metal loss (2.16% Li, <0.31% Ni/Co/Mn/Al) after 15 min of agitation [44]. Mechanistic studies revealed that CA-derived H^+^ ions attacked the aluminum oxide surface layer, disrupting PVDF–aluminum hydrogen bonding while forming a protective AlF_3_ passivation layer through reaction with LiF residues. Zhu introduced a thermal–chemical protocol combining CaCl_2_ (1:6 mass ratio) with microwave heating at 723 K (450 °C) under air atmosphere [13]. The resulting decomposition products (H_2_O vapor, HCl gas) facilitated PVDF degradation and active material release, with high-temperature Li^+^ diffusion enabling HCl-mediated conversion to soluble LiCl. This method achieved 93.4% stripping yield through weakened material–substrate adhesion. Fu et al. implemented a supercritical carbon dioxide (SC-CO_2_) system with dimethyl sulfoxide (DMSO) cosolvent at 70 °C and 80 bar pressure [42]. The supercritical fluid’s solvation power, synergized with DMSO’s polarity, disrupted PVDF intermolecular interactions, enabling selective binder dissolution and aluminum foil separation. Subsequent filtration yielded 96.7% active material recovery with minimal contamination, while PVDF precipitation was achieved through solvent cooling [42]. This approach demonstrates the potential of green chemistry principles in advanced battery recycling processes.

The calcination methodology for cathode material separation has evolved into two principal pathways: conventional high-temperature processes and emerging low-temperature catalytic techniques. While traditional methods achieve effective material dissociation through thermal decomposition mechanisms, they inherently exhibit limitations including excessive energy consumption, prolonged operational cycles, and structural degradation risks. In contrast, innovative catalytic approaches leverage temperature-controlled reactions to enable efficient energy utilization, preserve material integrity, and minimize metallic impurity contamination during active material detachment processes.

The traditional calcination method enables efficient cathode material separation through thermal decomposition of binders and organic components, though this approach exhibits notable drawbacks including high energy consumption, prolonged processing cycles, and risks of material degradation. Zhang demonstrated a controlled thermal protocol using a muffle furnace (KSL-1100 X), employing a two-stage nitrogen purge (250 mL/min initial flow >99.999% purity for 30 min, followed by 50 mL/min maintenance flow) during ramp-up to 600 °C (10 °C/min heating rate) to ensure complete binder/separator residue decomposition [51]. Wang combined solvent pre-treatment with thermal purification, initially removing partial PVDF via NMP ultrasonication before air calcination at 750 °C for 5 h to eliminate residual binders/organics, yielding purer LiCoO_2_ phases [53]. Jena implemented a hybrid approach, first thermally treating cathode materials at 700 °C for 5 h followed by 4 h NMP immersion and ultrasonic delamination, achieving 89.1% active material recovery [33]. Modern advancements focus on energy-efficient catalytic calcination technologies operating at reduced temperatures. Gu reported an anaerobic catalytic low-temperature calcination protocol (pre-roasting stage) that selectively decomposes PVDF and electrolyte residues while disrupting aluminum–NCM adhesion through catalytic effects, enabling facile powder detachment [54]. This innovative approach integrates deep debinding capabilities, minimized metal loss, and environmental sustainability, representing a significant improvement over conventional high-temperature processes.

The solvent leaching methodology enables efficient active material–aluminum foil separation through chemically selective dissolution mechanisms, combining advantages of high cathode material recovery rates, solvent recyclability, and environmental compatibility. Zhang demonstrated this approach using sodium hydroxide leaching under optimized conditions (368.15 K, 4:1 liquid–solid ratio mL/g, 2% alkali concentration, 5 min reaction time), achieving aluminum content reduction to <0.1% in LiFePO_4_ cathode products [50]. Fu optimized a 0.075 M NaOH system with 20 mL/g liquid–solid ratio and 40 min agitation, obtaining 98.4% separation efficiency with only 21 ppm residual aluminum in recovered materials [26]. Innovative hybrid systems integrate deep eutectic solvents (DES) with microwave assistance to achieve energy-efficient active material stripping. Li reported a choline chloride–ethylene glycol (ChCl-EG, 1:2 molar ratio) DES system operating at 120 °C with 8 min microwave treatment, achieving 100% aluminum foil detachment efficiency while maintaining solvent reusability through simple replenishment protocols [40]. This configuration enabled complete material recovery even after nine recycling cycles, demonstrating exceptional operational stability and economic viability.

**Figure 3:**
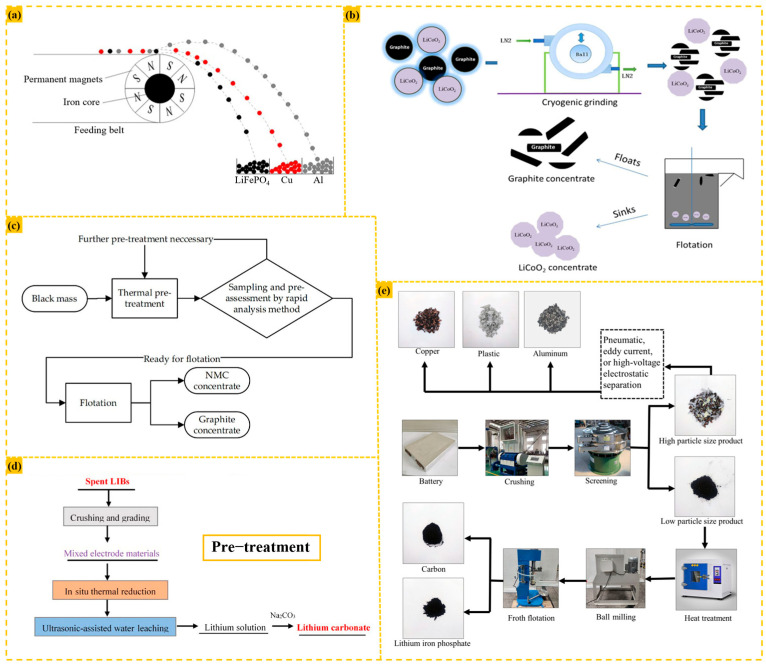
(**a**) Eddy current separation diagram [56]; (**b**) process of recovering LiCoO_2_ and graphite via cryogenic grinding and froth flotation [58]; (**c**) an example flow chart with the rapid analysis method integrated into the process [57]; (**d**) pre-treatment of spent LIBs by crushing and grading [30]; (**e**) overall recycling process of lithium iron phosphate battery [55].

In the laboratory, most positive active materials can be directly extracted from the positive electrode. However, in industrial production, mechanical crushing is typically used to separate electrode materials, current collectors, and metal casings. After crushing spent lithium-ion batteries, the positive and negative electrode materials become mixed. Consequently, various methods such as magnetic separation and flotation have been extensively explored to isolate positive active materials from this mixture, as illustrated in Figure 3.

Ding treated 10 g of water-leaching residue, composed of nickel and cobalt metals, manganese oxide, aluminum foil, copper foil, and graphite, using a Davis magnetic separator (xcgs-50) with a 300 rpm magnetic stirrer, 3000 OE magnetic field strength, and a feed particle size of −0.074 mm. The resulting magnetic fraction contained 26.34% nickel, 14.26% cobalt, and 28.30% manganese, with recovery rates of 92.57%, 94.16%, and 85.68%, respectively. Meanwhile, the recovery rates of aluminum and copper in the tailings were only 1.62% and 2.14% [30].

Wang heat-treated lithium iron phosphate electrode material at 450 °C for 20 min to decompose surface adhesives, exposing the hydrophilic LiFePO_4_ surface (reducing the contact angle from 80° to 26°) and dispersing agglomerated particles. Subsequent ball milling at 500 rpm for 3 min further broke down residual adhesives, enhancing carbon powder hydrophobicity (increasing the contact angle to 139°). Foam flotation at 2800 rpm and an aeration rate of 180 L/h yielded a lithium iron phosphate concentrate with 96.3% recovery and 93.5% grade, with minimal lithium loss of 67.83 mg/L [55].

Bi employed eddy current separation by placing crushed particles into an eddy current separator. With a magnetic roller speed of 800 rpm and a 1.5 m separation height, the distance between metal foil and LFP active material reached 101 mm, enabling non-overlapping separation. Copper and aluminum foils, being good conductors, experienced strong eddy current forces. When the vertical eddy current force exceeded gravity, they left the conveyor belt, driven horizontally by the magnetic force component, resulting in a longer flight distance. In contrast, the less conductive LFP active material, nearly unaffected by eddy currents, fell freely along the conveyor belt edge, with a shorter drop distance [56].

Qiu processed black material with a particle size <0.25 mm in a flotation machine. At a pulp concentration of 7.4%, stirring speed of 2000 rpm, and airflow rate of 40 L/h, the addition of 300 g/t collector and 150 g/t foaming agent produced foam products with 77% carbon content and 75% recovery. The pulp product contained 31% nickel and achieved 83% NMC purity, resulting in a 90% recovery rate of positive active materials [57].

Liu cryogenically ground electrode materials at 77 K for 9 min in a liquid nitrogen environment to embrittle and remove surface PVDF binders. Subsequent froth flotation using 250 g/t n-dodecane as collector and 180 g/t methylisobutyl carbinol (MIBC) as foaming agent, under conditions of 60 g/L pulp concentration, 1600 rpm stirring, and 0.7 L/min aeration, separated the anode material. This process yielded a LiCoO_2_ concentrate with a 91.75% grade and 89.83% recovery rate [58].

## 4. The Pyrometallurgical Technology for Recycling Spent Lithium-Ion Batteries

Pyrometallurgy in lithium-ion battery recycling is a process that uses high temperatures (typically above 1000 °C) to treat feedstock and extract metals or transform materials. Its core principle is harnessing high temperatures to induce phase transitions or decomposition reactions in substances. Based on temperature ranges, it can be categorized into high-temperature (1000–1450 °C), medium-temperature (500–800 °C), and low-temperature (<500 °C) processes. High-temperature processes employ traditional smelting for metal recovery but are energy-intensive and can lead to lithium loss. Medium-temperature processes utilize reduction or sulfidation roasting to enhance selective recovery efficiency [59]. Low-temperature processes are mainly used for pre-treatment to remove organics and prepare for subsequent hydrometallurgical processing.

**Figure 4:**
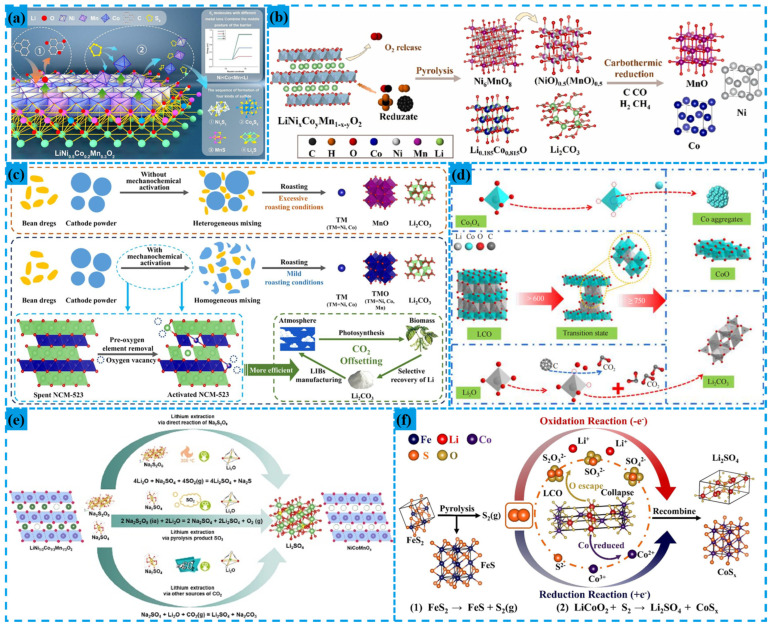
(**a**) Mechanism of selective sulfurization roasting [54]; (**b**) carbothermal reduction mechanism [15]; (**c**) schematic diagram of the action mechanism of MCA pre-treatment on the biomass reduction roasting [61]; (**d**) collapsing model of spent LCO cathodes at carbon reduction roasting [62]; (**e**) plausible conversion mechanism diagram of spent NCM cathode material in low-temperature reduction roasting [63]; (**f**) schematic diagram of the thermochemical reaction mechanism of the sulfidation roasting process [64].

Traditional high-temperature methods can recover multiple metals and effectively break down harmful substances. However, they also have drawbacks such as high energy consumption, stringent equipment requirements, and the tendency for lithium and other metals to remain in the slag, making recovery difficult [60]. To address these issues, innovative processes that lower roasting temperatures are being widely researched. These new approaches aim to reduce energy consumption while achieving high-efficiency lithium recovery and decreasing the high-temperature demands on equipment. Some of these methods are illustrated in Figure 4.

Sulfurization roasting is a common pyrometallurgical method valued for its high selectivity and efficient use of sulfur resources. For instance, Quan mixed FeS_2_ with lithium cobaltate at a mass ratio of 2.5:1 and roasted the mixture at 600 °C under a nitrogen flow of 30 mL/min for 2 h. This process yielded water-soluble Li_2_SO_4_ and insoluble Co and Fe sulfides. Above 420 °C, FeS_2_ decomposed into FeS and S_2_ gas; the S_2_ gas disrupted the layered structure of LiCoO_2_, releasing Li^+^ and forming a stable sulfate. Subsequent water leaching at 40 °C and 230 rpm for 1 h, followed by evaporation and concentration, achieved a Li^+^ concentration of 27.24 g/L. Adding Na_2_CO_3_ precipitated Li_2_CO_3_ with a purity exceeding 99.76% [64].

Gu blended NCM with carbon and sulfur at a mass ratio of 1:0.72:1.72 and roasted the mixture at 800 °C for 60 min, significantly lower than the traditional 1200 °C. The reactivity order of S and metals (Ni > Co > Mn > Li) generated sulfides like Ni_3_S_2_, Co_9_S_8_, and MnS, while Li converted to water-soluble Li_2_SO_4_/Li_2_S. Leaching at a solid–liquid ratio of 1:15 (g/mL) for 120 min at room temperature, followed by adding Na_2_CO_3_ and heating with stirring, produced Li_2_CO_3_ with a purity over 99%. The inclusion of carbon reduced the sulfidation temperature from 1200 °C to 800 °C. This was due to carbon’s preferential binding to O in NCM, lowering the metal’s oxidation state and promoting the sulfidation reaction [54].

The biomass-assisted method leverages renewable resources for roasting, offering environmental benefits and significant research potential. For example, Lin combined waste NCM cathode materials with soya bean dregs (BDs) at a mass ratio of 12.5%. They ball-milled the mixture at 500 rpm for 240 min with a ball-to-material ratio of 20:1. Roasting was carried out at 600 °C for 20 min under a nitrogen flow of 100 mL/min. Post-roasting, the products were ball-milled again at 500 rpm for 60 min and then leached in a water carbonate solution. This process achieved a lithium leaching rate of 97.2% [61].

Lu utilized the invasive plant Trifolium pratense as a raw material to reduce waste cathode powder via pyrolysis roasting. The process was conducted at 700 °C with a biomass ratio of 20% for 30 min. The roasted products were then mixed with water at a solid–liquid ratio of 1:30 for 60 min. This resulted in a lithium leaching rate of 94.18%. Subsequently, the filtrate was evaporated in an oven at 60 °C for 72 h to precipitate Li_2_CO_3_ solid [15].

The new pyrometallurgical process offers a high-efficiency, low-carbon approach for recycling spent lithium-ion batteries. By integrating strategies such as low-temperature roasting, physically enhanced leaching, and selective chemical modulation, it markedly cuts energy use, boosts lithium recovery, and improves environmental performance. For instance, Wei combined carbon reduction roasting with physically enhanced leaching. LCO and carbon black, mixed at a 1:0.15 mass ratio, were stirred at 2800 r/min for 3 min to ensure homogeneity. Roasting at 750 °C for 60 min under nitrogen caused LCO to decompose into Li_2_O and Co_3_O_4_. Co_3_O_4_ reduced to CoO and Co-metal, while Li_2_O reacted with CO_2_ to form water-soluble Li_2_CO_3_. The roasted product was wet ground to 15.81 μm, then ultrasonically crushed to 8.81 μm to free encapsulated lithium. Leaching at a solid–liquid ratio of 1:25 (g/mL), 500 r/min agitation, and 80 °C for 1.5 h dissolved Li_2_CO_3_, with cobalt species forming insoluble residues. Evaporation and Na_2_CO_3_ precipitation yielded Li_2_CO_3_ with a 99.10% recovery rate and 99.55% purity [62].

Yang roasted cathode material mixed with Na_2_S_2_O_8_ and Na_2_SO_4_ (1:1:1 mass ratio) at 300 °C for 30 min. Na_2_S_2_O_8_ decomposed, releasing SO_2_ and O_2_, which facilitated Li^+^ release and transition metal oxide formation. Na_2_SO_4_ stabilized the system, curbing SO_2_ emissions and promoting Li_2_SO_4_ generation. Leaching at 50 g/L solid–liquid ratio for 60 min at room temperature dissolved Li_2_SO_4_, while Ni, Co, and Mn remained as oxides in the residue. After adjusting the leachate pH to 12.0 to remove impurities, Na_2_CO_3_ precipitation and evaporation produced lithium carbonate with >99.3% purity. The high reactivity of Na_2_S_2_O_8_ lowered the lithium-ion conversion temperature from 600 °C to 300 °C, cutting energy use by 47.2% [63].

## 5. The Hydrometallurgical Technology for Recycling Spent Lithium-Ion Batteries

### 5.1. Acid Leaching System

Acid leaching systems in hydrometallurgical processes solubilize target metals as ionic species through acidic dissolution. This method offers notable advantages including high extraction efficiency, selective metal recovery, eco-compatibility, and operational adaptability. However, challenges persist with equipment corrosion, complex wastewater management, impurity interference, and elevated reagent costs due to acid consumption. The leaching systems can be categorized based on acid type: inorganic acid systems primarily employ sulfuric acid (H_2_SO_4_) or hydrochloric acid (HCl) as principal leaching agents, while organic acid systems utilize carboxylic acids such as citric acid (C_6_H_8_O_7_) or oxalic acid (C_2_H_2_O_4_) for selective metal complexation.

**Figure 5:**
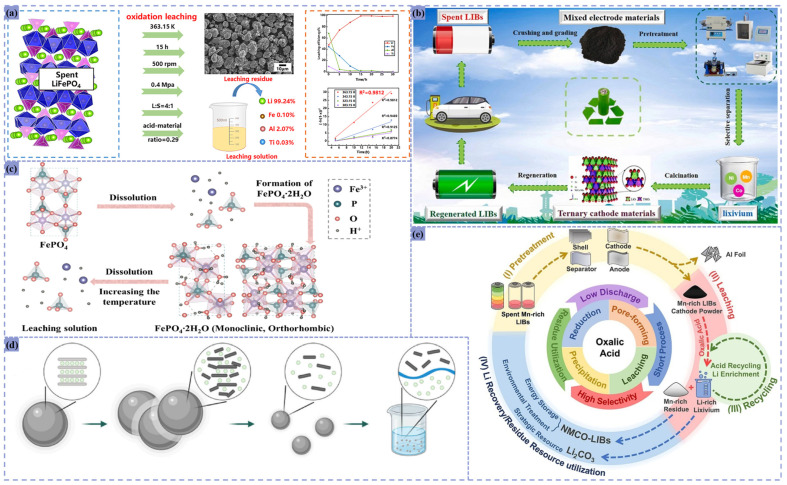
(**a**) Process of oxidative leaching and metal recovery from spent LiFePO_4_ batteries [50]; (**b**) sustainable regeneration process from spent to regenerated lithium-ion batteries [30]; (**c**) leaching mechanism of iron phosphate residue [65]; (**d**) schematic diagram of the combination approach for selective lithium leaching [51]; (**e**) sustainable recycling of spent ternary LIBs [66].

Inorganic acid leaching systems achieve efficient metal dissolution and selective separation through acidic environments, offering low operational costs despite equipment corrosion risks and environmental concerns. As illustrated in Figure 5, single-acid leaching protocols demonstrate exceptional performance across diverse feedstocks.

Yang achieved 99.2% Fe and 98.0% P recovery through iron phosphate slag pre-treatment combined with H_3_PO_4_ leaching under optimized conditions: 6 mol/L acid concentration, 7.5 mL/g liquid-to-solid ratio, 75 °C, and 180 min reaction duration [65]. Zhang subjected thermally reduced calcined ternary lithium-ion battery cathode material NCM, LiCoO_2_, and LiNiO_2_, to mechanical activation via planetary ball milling operated at 10 Hz for 30 min. This was followed by sulfuric acid leaching (2 mol/L H_2_SO_4_) at 35 °C with a solid-to-liquid ratio of 100 g/L for 5 min. The process achieved 91.6% lithium leaching efficiency with 92.2% selectivity for NCM materials, while LiCoO_2_ and LiNiO_2_ demonstrated 95.8% and 100% lithium extraction, respectively [51].

Pressurized oxidative H_2_SO_4_ leaching of decontaminated LiFePO_4_ cathodes under optimized conditions (500 rpm, 15 h, 363.15 K, 0.4 MPa, 4:1 liquid–solid ratio, 0.29 acid ratio) achieved 99.24% Li leaching with minimal Fe/Al/Ti contamination (0.10%, 2.07%, 0.03% respectively) [50]. In Ding’s research, magnetic composite particles (e.g., Ni/MnO, Co/MnO) applied to magnetic separation concentrates demonstrated 92.57% Ni, 94.16% Co, and 85.68% Mn recoveries via 2 mol/L H_2_SO_4_ leaching at 50 °C, producing leachates containing 13.08 g/L Ni, 7.10 g/L Co, and 14.06 g/L Mn with total impurity levels (Al, Cu, Fe) below 0.05 wt% [30].

Liang et al. employed OA for the treatment of spent NCM electrode materials under optimized conditions: 80 °C reaction temperature, 20-minute reaction duration, and 20 g/L solid-to-liquid ratio. The process achieved 95.7% lithium leaching efficiency with 90.0% selectivity. This approach enables efficient and selective lithium recovery by strategically utilizing OA’s acidity, reducing capability, and the solubility product differences among metal oxalates [66].

**Figure 6:**
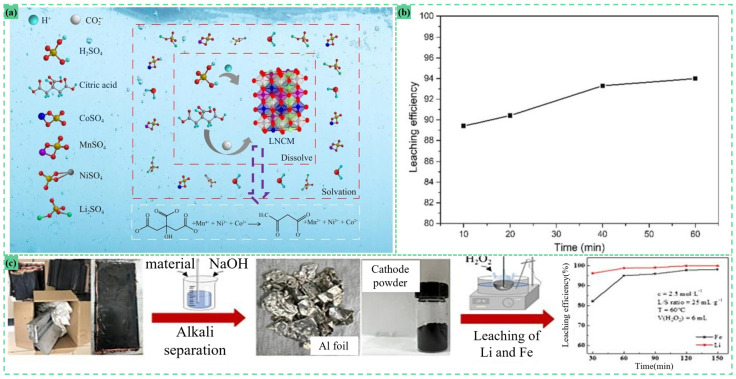
(**a**)Synergistic leaching mechanism of H_2_SO_4_ and C_6_H_8_O_7_ [68]; (**b**) time-dependent leaching efficiency of cobalt at 1 M HNO_3_ containing 10% H_2_O_2_ [67]; (**c**) a process for efficient leaching, recovery, and regeneration of lithium and iron from waste lithium iron phosphate cathode materials [26].

Conventional single-acid leaching systems often suffer from low efficiency, poor selectivity, excessive reagent consumption, and secondary pollution risks. However, the synergistic combination of acid and reductant significantly enhances metal recovery, reduces impurity interference, and minimizes environmental impact. These technologies have garnered extensive research and industrial application, as demonstrated in Figure 6.

For instance, Fu et al. demonstrated that a H_2_SO_4_-H_2_O_2_ system achieved 98.79% Li and 94.97% Fe recoveries from LiFePO_4_ cathode powder under optimized conditions: 2.5 M H_2_SO_4_, 6 mL H_2_O_2_, 25 mL/g solid-to-liquid ratio, 60 °C for 60 min [26]. Similarly, LCO cathode materials showed 94% Li extraction when leached with 1 M HNO_3_ and 10 vol% H_2_O_2_ at room temperature (1:50 solid–liquid ratio, 60 min). The redox activity of H_2_O_2_, which reduces Co^3+^ to Co^2+^, facilitates matrix dissolution [67].

Ren demonstrated a synergistic leaching approach using sulfuric acid (1.16 mol/L) and citric acid (15 wt%) for spent NCM recovery. Operating at 83℃ for 120 minutes with a 40 g/L solid-to-liquid ratio, the process achieved exceptional metal recovery: 99.08% for lithium, 98.76% for nickel, 98.33% for cobalt, and 97.63% for manganese. The leaching en-hancement mechanism involves dual effects: citric acid’s carboxyl groups stabilize diva-lent metal ions (Mn^2+^, Ni^2+^, Co^2+^) through chelate complexation, preventing precipitation while maintaining solution stability. Simultaneously, H^+^ ions released from citric acid dissociation synergize with sulfuric acid to intensify proton attack on the layered oxide structure, accelerating lithium release [68].

**Figure 7:**
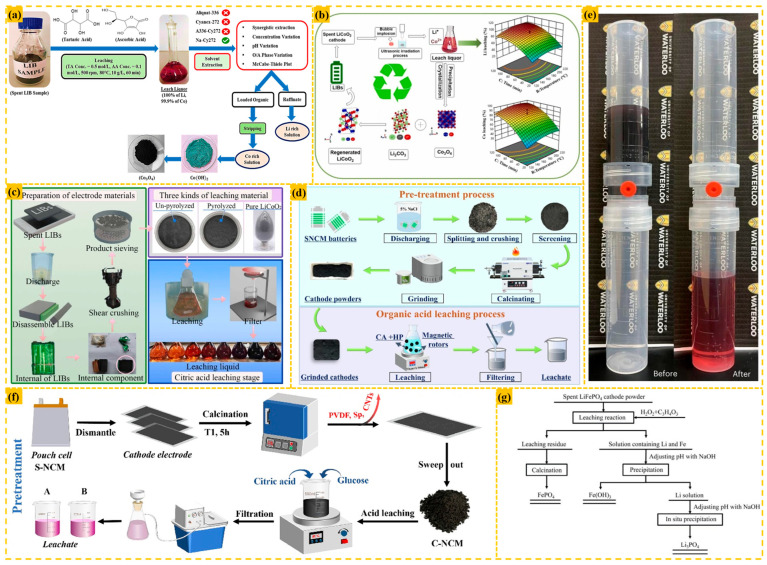
(**a**) Synergistic approach for leaching and separation of metals from spent LIBs [16]; (**b**) ultrasound-assisted recycling of valuable metals from spent Li-ion batteries [46]; (**c**) experimental flow chart [43]; (**d**) process flow diagram of pre-treatment and organic acid leaching for SNCM batteries [72]; (**e**) filtration process by a vacuum-assisted DigiFILTER assembly [69]; (**f**) detailed pre-treatment procedure for spent LIBs [71]; (**g**) process flow of recovering valuable metal from spent LiFePO_4_ cathode powder [70].

Organic acid leaching systems offer environmental compatibility, high selectivity, and mild operational conditions, eliminating the need for strong acids or elevated temperatures. This makes them particularly suitable for complex material matrices, though operational costs remain relatively high. As illustrated in Figure 7, various organic acid formulations demonstrate exceptional performance in battery recycling applications.

Leaching LiCoO_2_ anode materials with 1 M citric acid and 0.2 M ascorbic acid at 70 °C for 30 min achieved 99.17% Li and 99.31% Co recoveries [43]. Sahu et al. processed unsorted LiCoO_2_ electrodes (<25 μm particle size) using 0.5 M tartaric acid and 0.1 M ascorbic acid at 80 °C (10 g/L solid–liquid ratio, 500 rpm agitation, 60 min), achieving 100% Li and 99.9% Co extraction without Cu/Al dissolution [16]. Ultrasound-assisted gluconic acid leaching of LiCoO_2_ powder under 40% ultrasonic power, 0.4 M gluconic acid, 180 °C, and 80 min yielded 98.59% Li and 97.17% Co recoveries, with ultrasonic cavitation enhancing matrix disruption and gluconic acid providing H^+^ coordination for metal dissolution [46].

For LiCoO_2_ recycling, 2.5 M acetic acid with 2 vol% H_2_O_2_ at 70 °C (10 g/L slurry density, 1 h) achieved >95.6% Co and >97.4% Li recoveries, with H_2_O_2_ facilitating oxidative metal release while retaining Al/Cu impurities in solid phase [69]. Discarded LiFePO_4_ cathodes leached in 3.0 M pyruvic acid with 2 mL H_2_O_2_ at 80 °C (0.1 g/mL solid–liquid ratio, 20 min) reached 96.56% Li recovery [70].

Zhou employed citric acid as an extractant for spent NCM cathode material recovery. Under optimized leaching conditions (20 g/L solid-to-liquid ratio, 1.5 mol/L acid concentration), the process achieved 99.2% Li, 99.5% Ni, 99.4% Co, and 99.7% Mn leaching efficiencies. To maintain stable metal ion dissolution and prevent hydroxide precipitation, the pH must be precisely controlled between 1.5 and 3.0 during operation [71]. Similarly, citric acid (4 M) with 2 vol% H_2_O_2_ at 80 °C (20 g/L solid–liquid ratio, 70 min) extracted 96.69% Ni, 95.38% Co, 97.64% Mn, and 98.72% Li from spent NCM batteries [72]. These systems leverage organic acid complexation and proton exchange mechanisms to achieve selective metal recovery while minimizing secondary waste generation.

### 5.2. Deep Eutectic Solvent Leaching System

Deep eutectic solvents (DESs) are gaining attention as a green and efficient leaching medium due to their low volatility, high stability, and recyclability, offering a more environmentally friendly alternative to conventional strong acids and alkalis. However, challenges such as complex solvent regeneration processes and the thermal instability of certain components at elevated temperatures still need to be addressed.

**Figure 8:**
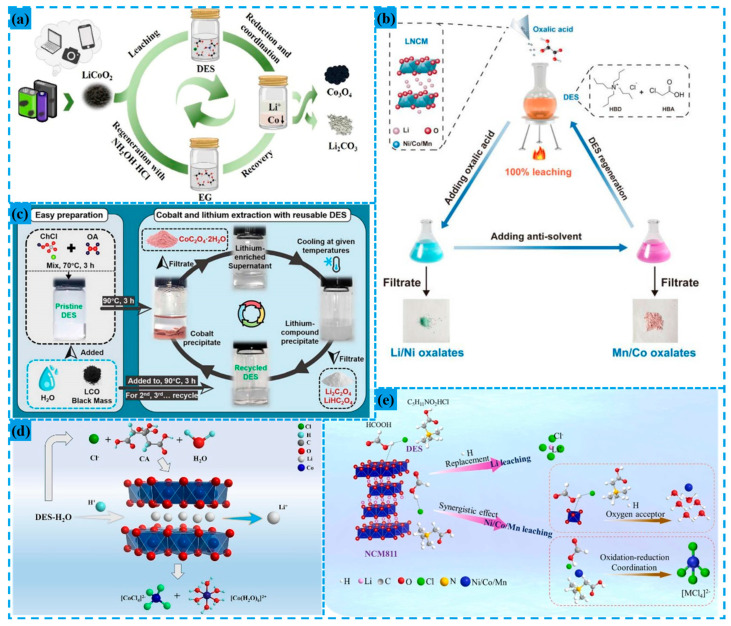
(**a**) Process of valuable metals recycling from spent lithium-ion battery cathodes using deep eutectic solvent [17]; (**b**) a proposed flowchart for recovering LNCM using the DES [73]; (**c**) schematic procedure showing the closed-loop recycling of LCO based on a reusable DES [74]; (**d**) mechanism of critical metals recycling from LiCoO_2_ batteries using hydrated deep eutectic solvents [75]; (**e**) plausible pathways for critical metal leaching from NCM811 cathode materials via BeCl:FA DES [76].

Common DES compositions include combinations of organic salts (e.g., choline chloride) whit metal salts (e.g., FeCl_3_), mixtures of hydrated metal salts (e.g., ZnCl_2_·2H_2_O) with organic salts, and blends of metal salts (e.g., LiCl) with hydrogen bond donors (HBDs). Nonionic DESs, such as menthol combined with thymol, are also used. Additionally, DESs formed from organic salts and HBDs with hydroxyl or carboxylic acid groups (e.g., ethylene glycol or glycerol) are particularly common in lithium-ion battery recycling and have attracted significant research interest. Some methods for recycling waste lithium-ion batteries using DES leaching systems are illustrated in Figure 8.

The application of DES in treating various lithium batteries offers distinct advantages. For instance, when processing LCO batteries, DES leverages an acidic environment to disrupt the LiCoO_2_ structure, promotes stable complex formation between Cl^−^ and Co^2+^, and utilizes the synergistic effect of reducing agents to achieve efficient leaching of lithium and cobalt. Integrating solvent regeneration technology ensures cycling stability, while demonstrating high selectivity toward single-metal systems and environmental benignity.

Tian et al. employed a DES composed of ethylene glycol (EG) and hydroxylamine hydrochloride (NH_2_OH·HCl) as the leaching agent for lithium cobaltate battery cathode materials. At 80 °C for 8 h with a solid–liquid ratio of 1:12, this process achieved leaching efficiencies of 99.7% for lithium and 88.0% for cobalt [17]. Wang et al. utilized choline chloride–citric acid (ChCl/CA)-hydrated DES (25.7 wt% water) to leach lithium and cobalt. Under conditions of 4 h leaching time, 120 °C temperature, and a liquid-to-solid ratio of 50 mL/g, they obtained leaching rates of 97.6% for Co and 100% for Li. This performance resulted from water reducing DES viscosity, H^+^ disrupting the LiCoO_2_ structure, Cl^−^ forming [CoCl_4_]^2−^ complexes with Co^2+^, and citric acid (CA), providing an acidic-reducing environment. After three DES solvent cycles, the cumulative leaching rate decrease was approximately 10%, recoverable by CA supplementation [75]. Hu et al. used a 1:1 molar ratio ChCl:oxalic acid (OA) DES as the leaching agent for LCO cathode materials at 90 °C for 3 h, achieving 92% lithium leaching rate. By evaporating water and ethanol from the filtrate, the DES was recovered and recycled three times, maintaining stable leaching efficiency (92% Li leaching, Co residue < 3%) [74].

The use of DES for treating ternary lithium batteries offers notable advantages: DES can efficiently dissolve multiple metals (Li, Ni, Co, Mn) through the synergistic effects of an acidic environment and Cl^−^ coordination, while their low viscosity enhances mass transfer. Selective precipitation technology enables stepwise metal recovery, and solvent regeneration allows recycling, thus demonstrating adaptability to complex ternary systems, environmental compatibility, and high metal recovery efficiency.

For example, Zhang et al. employed a DES with a 3:1 molar ratio of monochloroacetic acid (MCA) and tetrabutylammonium chloride (TBAC) to treat ternary lithium battery cathode materials (LNCM). At 100 °C for 7 h (solid–liquid ratio 15 g/L, with 0.4 g/g oxalic acid as a reductant), complete dissolution of Li, Ni, Co, and Mn was achieved. By sequentially adding oxalic acid, they selectively precipitated Li/Ni (87.1% and 99.4% recovery, respectively) and Co/Mn (100% and 80% recovery, respectively) through steps involving oxalic acid addition (0.8 g/g LNCM, 60 °C for 2.5 h) and aqueous oxalic acid (0.12 mol/L, same volume as DES). The DES was regenerated via vacuum water evaporation, maintaining 100% metal dissolution efficiency over 10 recycling cycles [73].

Liu et al. used a low-viscosity DES composed of betaine hydrochloride (BeCl) and formic acid (FA) at a 1:9 molar ratio to leach NCM811 cathode material at 140 °C for 360 min (solid–liquid ratio 20 g/L). This process achieved leaching rates of 98.03% for Li, 92.35% for Ni, 94.03% for Co, and/ 96.01% for Mn. DES regeneration via vacuum evaporation removed water, sustaining leaching efficiency across 10 cycles. The system leverages FA to provide H^+^ for lattice disruption and reduction in highly oxidized metals, while Cl^−^ from BeCl coordinates with metal ions, enabling synergistic dissolution of valuable metals [76].

### 5.3. Novel Leaching Systems for Lithium-Ion Battery Recycling

**Figure 9:**
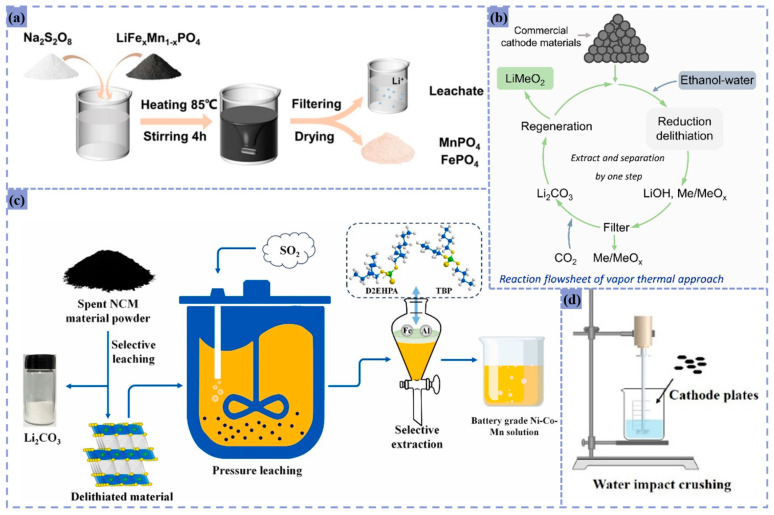
(**a**) Schematic diagram for leaching of the spent LMFP powders [77]; (**b**) the recovery flow sheet of ethanol vapor thermal for recycling degraded LCO cathode materials [78]; (**c**) sketch of the experimental procedures in this study [79]; (**d**) water-impact crushing leaching of cathode plates [48].

The recovery of ternary lithium batteries involves substantially greater technical challenges compared to lithium cobalt oxide (LCO) and lithium iron phosphate (LFP) batteries. These complexities arise from multiple factors: the diversity of metal components within the battery chemistry, inherent structural stability that complicates material separation, stringent environmental protection standards, and regulatory hurdles in market implementation. To address these challenges, innovative leaching systems have been proposed specifically for ternary lithium battery recycling, including selective oxidative leaching and pressurized reductive acid leaching techniques. Similar advancements have emerged for other lithium battery variants, with all methodologies sharing a common objective: to enhance recovery efficiency while simultaneously strengthening environmental stewardship, as illustrated in Figure 9.

In the recovery of ternary lithium batteries, Qing employed SO_2_ gas for pressurized leaching of pretreated NCM cathode materials. Under a SO_2_ partial pressure of 0.25 MPa, 70 °C, and a liquid–solid ratio of 150 g/L, a 60 min reaction achieved elemental leaching efficiencies of 99.6% for Ni, 99.3% for Co, and 99.6% for Mn, with a total metal concentration of 140 g/L in the leachate. Subsequent addition of H_2_SO_4_ and lithium removal agents further utilized residual SO_2_, increasing the overall metal recovery rate to over 98% [79].

Xie conducted a stirred reaction of LiFe_x_Mn_1−x_PO_4_ (LMFP) powder with Na_2_S_2_O_8_ at 85 °C for 20 min, achieving a lithium leaching rate of 98.84% under a liquid–solid ratio of 5:1. Iron and manganese leaching remained minimal at 0.01% and 0.67%, respectively. This selectivity stems from Na_2_S_2_O_8_ acting as a strong oxidizing agent, converting Fe^2+^ and Mn^2+^ in LMFP to Fe^3+^ and Mn^3+^. This disrupts the lattice structure to release Li^+^, while iron and manganese remain as insoluble phosphates and lithium dissolves as Li_2_SO_4_ [77].

Yuan investigated a two-stage leaching process for magnetized roasted ternary cathode material (LiNi_0.5_Co_0.2_Mn_0.3_O_2_). The first stage used room-temperature water leaching (solid–liquid ratio 1:5, pH = 7, 60 min stirring), achieving a 40.80% lithium leaching rate. The second stage involved carbothermal reduction of residual cathode material at 700 °C with 15% toner, followed by water leaching to extract the remaining lithium, yielding a total leaching rate of 95.30% [48].

Regarding lithium cobaltate batteries, Qu explored a novel leaching method in a 50 mL closed reactor, using a mixed ethanol–water vapor solution to leach LCO powder. Reacting at 370 °C for 2 h or 400 °C for 0.8 h achieved a 99.5% Li leaching rate. Here, ethanol acts as a reducing agent, converting high-valence transition metal oxides to lower valence states, allowing lithium to dissolve in water as LiOH [78].

### 5.4. Recovery of Metal Ions from Leachate Solutions

**Figure 10:**
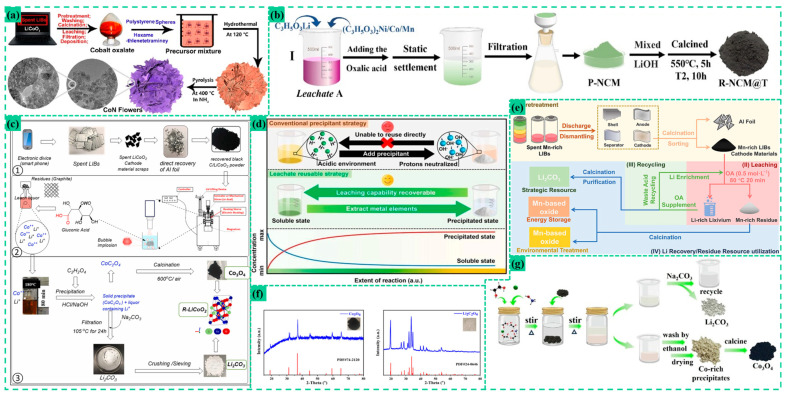
(**a**) Schematic for the complete processes of recycling Co in LCO LIBs and the controllable preparation of 3D flower-like CoN NFs [53]; (**b**) the detailed post-treatment procedure for spent LIBs and regeneration technology [71]; (**c**) proposed flowsheet of the direct recycling route of spent lithium-ion batteries [46]; (**d**) the reversible regulation of solubility could enable the reversible control for the ratio of the metal elements in the soluble state and precipitated state [74]; (**e**) illustration of the resource utilization process of spent ternary Mn-rich LIBs [66]; (**f**) XRD and SEM images of Co_3_O_4_ and Li_2_C_2_O_4_ precipitates [75]; (**g**) overview of the process for the recovery of value metals by dissolution from LiCoO_2_ [17].

Acid leaching, DES, and novel leaching systems effectively dissolve valuable metals from solid cathode materials into solution. Once dissolved, selectively recovering these metal ions becomes critical. Conventional methodologies for valuable metal recovery from leachates primarily involve chemical precipitation and physical property-based separation techniques, which enable selective isolation of target metals through differential physical/chemical behavior. Representative recovery methods are illustrated in Figure 10.

The chemical precipitation method is widely used in metal recovery, typically involving pH adjustment or precipitant addition to form insoluble metal compounds. A common approach for lithium recovery is carbonate precipitation. For example, adding a 2.0 mol/L sodium carbonate solution at 2.5 mL/min to a 2.5 mol/L lithium chloride solution under 90 °C and 400 rpm stirring yields lithium carbonate with 85.72% recovery and 98.19% purity [80]. In another case, bubbling CO_2_ through an LiOH-rich solution for 2 h converts LiOH to Li_2_CO_3_ precipitate, which after filtration and 60 °C vacuum drying achieves >99% purity [78].

When processing leached mixtures, centrifugation separates the reaction products into a supernatant and a cobalt hydroxylamine complex. Adding Na_2_CO_3_ to the supernatant, stirring at 60 °C for 5 h, and subsequent centrifugation collects Li_2_CO_3_ precipitate. Washing with anhydrous ethanol and vacuum drying at 60 °C yields white Li_2_CO_3_ powder [17]. Alternative precipitation methods have also been explored: adjusting leachate pH to 12.0 with NaOH and stirring at 80 °C for 2 h forms Li_3_PO_4_ precipitate with 96.5 wt% purity [70]. Adding oxalic acid to the leachate, precipitating at 20 °C for 30 min, and calcining at 500 °C for 6 h produces cobalt oxalate (CoC_2_O_4_·2H_2_O) and cobalt oxide (Co_3_O_4_, black powder) with 98.02% purity. Evaporating water and adding anhydrous ethanol to the leachate yields lithium oxalate (Li_2_C_2_O_4_) with 99.14% purity [75].

Stepwise precipitation enables sequential metal recovery by leveraging solubility differences. For instance, adjusting leachate pH to 12, adding saturated Na_2_CO_3_, and stirring at 85 °C for 1 h yields 99% white Li_2_CO_3_. Lowering pH to 8.5 and adding oxalic acid forms CoC_2_O_4_·2H_2_O, which, calcined at 600 °C, gives 98.8% black Co_3_O_4_ powder [46]. Selective cobalt oxalate precipitation at adjusted pH achieves 95.6% Co recovery with >99.69% CoC_2_O_4_ purity [53]. Precipitating metal hydroxides by adjusting pH to 10 with NaOH, followed by 300 °C calcination for 120 min, yields Co_3_O_4_ exceeding 98.8% purity [66].

In citric acid leachates, adding oxalic acid at pH 2.5–3.0, gentle heating to 50 °C, and rapid stirring exploits solubility product differences to selectively precipitate Ni^2+^, Co^2+^, and Mn^2+^ as oxalate precursors (p-NCM). After precipitation, adjusting filtrate pH to 1.5–2.0 regenerates citric acid for recycling, reducing reagent consumption [71]. Chen’s method involves room-temperature water soaking of thermochemical reduction products (20 g/L slurry density, 3 h) to selectively extract Li_2_CO_3_, which after evaporation yields battery-grade lithium carbonate (99.99% purity). For water-leached residues, low-temperature roasted (300–500 °C) NiO, CoO, and MnO are leached with 0.55 M sulfuric acid at 50 °C (30 g/L slurry density, 30 min), achieving 97.46% Co, 95.84% Ni, and 91.28% Mn recovery. Adjusting leachate molar ratios enables coprecipitation of ternary precursor Ni_x_Co_y_Mn_1−x−y_(OH)_2_. High-temperature roasted (500–700 °C) residues first undergo magnetic separation for Ni/Co recovery, followed by mild acid leaching of MnO to obtain MnSO_4_, enabling quality recovery of transition metals [81].

Beyond chemical precipitation, physical separation and thermal treatment techniques provide alternative methods for separating valuable metals. These approaches feature simple, environmentally friendly processes that utilize physical mechanisms—including solubility product adjustment, solubility differences, and thermal decomposition—without relying on traditional chemical precipitants.

For instance, leachates can selectively precipitate metals through dynamic regulation of the solubility product (Ksp). By adding 50 wt% deionized water, the solution is diluted, altering the ligand environment and prompting Co^2+^ to combine with C_2_O_4_^2−^, forming cobalt oxalate precipitate. Cooling the filtrate to −30 °C reduces the solubility of Li_2_C_2_O_4_ and LiHC_2_O_4_, yielding lithium oxalate precipitate. Notably, this method avoids conventional precipitants while maintaining leachate reusability [74].

Cobalt-hydroxylamine-chloride complex precipitate undergoes a post-treatment process: washing with anhydrous ethanol, drying in a 60 °C vacuum oven, and calcining in a muffle furnace at 600 °C for 4 h. This decomposes the complex into Co_3_O_4_, which is then ground to produce black Co_3_O_4_ powder. This process achieves lithium and cobalt recoveries of 99.7% and 88.0%, respectively [17].

Solvent dissolution and filtration have also been used to separate Li_2_C_2_O_4_ from mixed precipitates. Zhang dissolved Li_2_C_2_O_4_ using 1.5 times the minimum water required for full dissolution. Filtration separated NiC_2_O_4_·2H_2_O precipitate from the Li_2_C_2_O_4_ aqueous solution, and subsequent evaporation of the solution yielded pure Li_2_C_2_O_4_ product [73].

## 6. Novel Green and High-Efficiency Recovery Methodology

**Figure 11:**
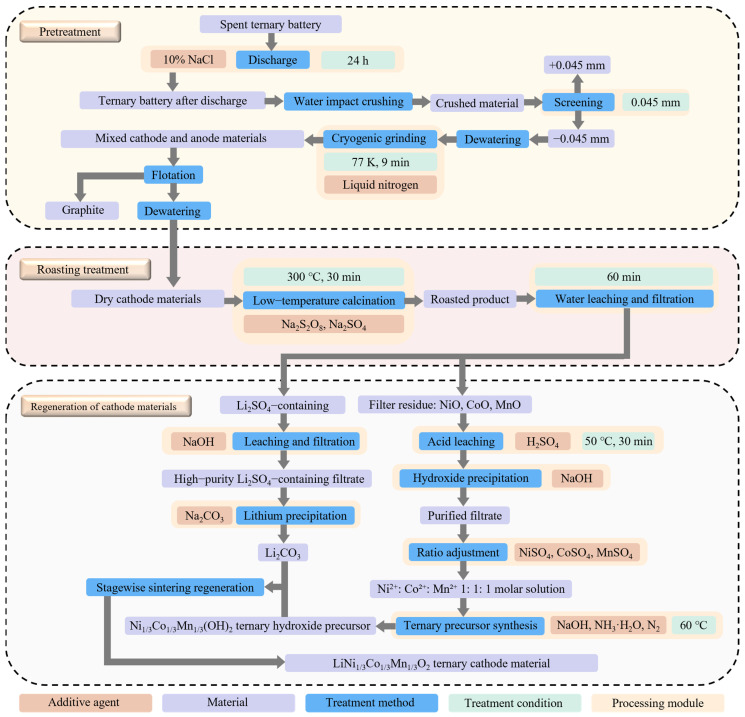
An innovative recycling process for spent ternary lithium batteries.

Through recycling treatment of ternary lithium-ion batteries, renewed ternary cathode materials can be generated, thereby realizing the closed-loop recycling of ternary lithium-ion batteries and establishing a sustainable material circulation pathway. The core stages of this process primarily encompass pre-treatment, low-temperature roasting, and utilization of the roasted products. The novel approach is illustrated in Figure 11.

The pre-treatment stage aims to isolate high-purity cathode materials for subsequent processing. Waste lithium-ion batteries are first discharged in a 10% NaCl solution for 24 h at 35 °C to ensure thorough discharge. Water-impact crushing follows, preventing cathode material and conductive agents from escaping as dust while preserving the cathode surface integrity.

After crushing, the material is sieved through a 0.045 mm mesh. Crushed battery shells remain on the sieve, while cathode, anode, and collector materials pass through to the undersize fraction. This undersize material is dehydrated in a drying oven, then cryogenically milled at 77 K for 9 min in a liquid nitrogen environment. This process embrittles PVDF binder, facilitating its detachment from the cathode material.

Following binder removal, the electrode mixture undergoes froth flotation. At a slurry concentration of 60 g/L, with 1600 rpm stirring and 0.7 L/min air flow, 250 g/t n-dodecane (collector) and 180 g/t MIBC (foaming agent) are added. This separates anode materials, after which the cathode fraction is dried to yield high-purity cathode electrode materials.

Following the isolation of high-purity cathode material, valuable metals are extracted through a thermal treatment process. The cathode material is first mixed with Na_2_S_2_O_8_ and Na_2_SO_4_ at an optimal mass ratio of 1:1:1. This mixture is then loaded into a high-temperature furnace and roasted isothermally at 300 °C for 30 min. During roasting, a chemical transformation occurs: elemental lithium converts to water-soluble lithium sulfate, while transition metals (Ni, Co, Mn) form water-insoluble oxides. By using this specific reagent ratio, the roasting temperature is substantially lowered, achieving both environmental benefits and effective chemical separation of lithium from transition metals. This creates a critical foundation for subsequent steps: dissolving lithium sulfate in water to isolate lithium and processing the transition metal oxides for individual recovery.

Following cooling of the roasted products, lithium is first separated via water leaching. The roasted material is mixed with deionized water at a 50 g/L solid–liquid ratio and leached for 60 min at room temperature under stirring. This step exploits the significant solubility difference between lithium sulfate (highly water-soluble) and transition metal oxides (NiO/CoO/MnO, poorly soluble) to achieve efficient solid–liquid separation of lithium from Ni-Co-Mn.

Gentle mechanical stirring ensures complete dissolution of roasted Li_2_SO_4_ into the liquid phase, while dense metal oxide particles remain as slag. This achieves a lithium leaching rate exceeding 98%. After initial filtration, the leachate is purified via stepwise alkaline precipitation: a 20% NaOH solution is slowly added using a peristaltic pump, adjusting the system pH to 12.0 under 200 rpm stirring. Under these conditions, trace co-soluble impurities (e.g., Fe^3+^, Al^3+^) precipitate as hydroxides, while Li^+^ remains in solution due to the high solubility of LiOH (12.8 g/L at 20 °C).

The clarified filtrate then undergoes lithium precipitation using a 300 g/L Na_2_CO_3_ solution as the precipitant. Leveraging the sharp solubility decline of Li_2_CO_3_ in alkaline conditions (<0.1 g/L at 25 °C, pH > 12), Li^+^ rapidly precipitates as white Li_2_CO_3_ crystals. Stirring at 150 rpm for 30 min promotes uniform particle growth and minimizes impurity adsorption. Finally, the slurry is centrifuged, washed three times with deionized water, and concentrated in a vacuum evaporator at ≤80 °C, yielding Li_2_CO_3_ with ≥99.3% purity.

Filter slag containing NiO, CoO, and MnO undergoes acid dissolution as the first step. The slag is mixed with H_2_SO_4_ solution and reacted at 50 °C for 30 min. This temperature ensures complete reaction between the metal oxides and sulfuric acid, dissolving Ni, Co, and Mn into the leachate efficiently while minimizing energy consumption and side reactions. Upon completion, the solution contains Ni^2+^, Co^2+^, Mn^2+^, and trace impurity ions (e.g., Al^3+^, Cu^2+^, Fe^3+^).

The solution pH is then adjusted to 4.8 using 4 M NaOH, inducing hydroxide precipitation of impurity ions. Filtration removes these solids, purifying the solution and reducing interference in subsequent steps. The impurity-free filtrate is analyzed via inductively coupled plasma optical emission spectrometry (ICP-OES) to determine initial Ni^2+^, Co^2+^, and Mn^2+^ concentrations. Based on these results, the ion ratios are adjusted to 1:1:1 by adding pure NiSO_4_, CoSO_4_, and MnSO_4_ solutions, establishing the stoichiometric foundation for ternary precursor co-precipitation.

The ratio-adjusted solution is transferred to a corrosion-resistant stainless-steel reactor. During the reaction, 2 M NaOH and 1 M ammonia are added simultaneously while controlling the pH at 10.5, maintaining 60 °C, and passing nitrogen to prevent metal ion oxidation. A high-speed mechanical stirrer (1300 rpm) ensures uniform mixing and full component contact, yielding the spherical hydroxide precursor Ni_1/3_Co_1/3_Mn_1/3_(OH)_2_. This precursor features uniform particle size and moderate specific surface area, facilitating subsequent cathode material synthesis via mixing with a lithium source and sintering—critical for enhancing electrochemical performance and processability, and serving as a key intermediate for high-value lithium-ion battery resource recycling.

The ultimate goal of electrode material recycling is to synthesize new electrode materials, thereby closing the loop of battery production—from manufacturing to end-of-life recycling and back to production. To achieve this closed-loop objective, this study designed a regeneration route to ensure the electrochemical properties of regenerated cathode materials closely mirror those of virgin materials.

First, the precursor Ni_1/3_Co_1/3_Mn_1/3_(OH)_2_ is mixed with Li_2_CO_3_ at a Li/Me molar ratio of 1.05, with a slight lithium excess to compensate for volatilization losses during high-temperature sintering—since part of the lithium is lost as a gas phase during this process. Homogenization is achieved via ball milling with anhydrous ethanol as the dispersant, yielding a uniformly gray powder to ensure intimate contact between raw materials.

The blended powder is loaded into an alumina crucible (high-temperature resistant and non-reactive with lithium) and subjected to staged sintering in a box-type muffle furnace. The first stage involves heating to 450 °C at 8 °C/min for 5 h of pre-sintering, designed to remove residual moisture, ammonia decomposition products, and other volatile compounds from the precursor. This step prevents particle rupture or pore formation caused by sudden gas release at high temperatures while promoting initial surface reactions between lithium carbonate and the precursor to form small composite oxide nuclei, laying the foundation for subsequent reactions. After pre-sintering, the powder contracts slightly, darkens in color, and becomes more compact.

In the second sintering stage, heating continues at the same rate to 900 °C, with a 20 h hold in an air atmosphere. The air provides stable oxidation conditions for cobalt and manganese ions, ensuring their participation in forming the layered structure as high-valent states (Co^3+^, Mn^4+^). At high temperatures, lithium carbonate decomposes into lithium oxide (Li_2_O) and releases carbon dioxide, enabling solid-phase diffusion between Li_2_O and the nickel/cobalt/manganese oxides formed by precursor dehydration. Lithium ions gradually intercalate into the transition metal oxide lattice, ultimately forming LiNi_1/3_Co_1/3_Mn_1/3_O_2_ with a hexagonal layered structure. The precursor’s spherical morphology is maintained during sintering, with particle size reducing to 8–10 μm due to thermal shrinkage, resulting in smooth, crack-free surfaces that enhance material tap density and electrode processability.

After sintering, the furnace power is turned off, and the powder is cooled to room temperature (approximately 12 h) to avoid particle cracking caused by thermal stress from rapid cooling. The cooled sintered blocks are gently ground in a ceramic mortar and sieved through a 100-mesh screen to remove agglomerates, finally yielding black spherical LiNi_1/3_Co_1/3_Mn_1/3_O_2_ powders.

## 7. Discussion

Spent LIBs contain abundant strategic metals like lithium and cobalt that warrant recovery, yet current recycling rates remain critically low. These spent batteries pose environmental risks due to toxic heavy metals and electrolyte leakage. Developing efficient recycling technologies to enhance metal recovery yields while ensuring process efficiency and environmental sustainability therefore holds dual significance for resource conservation and ecological protection.

(1) Waste lithium-ion batteries undergo mechanical separation processes based on component-specific physical properties, enabling efficient disassembly of valuable materials through sequential screening, magnetic separation, and froth flotation. Current collectors exhibit exceptional mechanical resilience during size reduction, resisting fragmentation below critical particle diameters. This allows gravitational screening to effectively isolate coarse-grained metallic foils from fine-grained active material powders. For the screened electrode fractions, flotation offers superior selectivity for cathode/anode separation compared to alternative methods, leveraging surface property differences between lithium-bearing cathode powders and graphite-based anode materials. Post-flotation concentration, various hydrometallurgical pathways can be implemented to recover strategic metals from the purified cathode streams.
(2) Pyrometallurgical processes encompass sulfurization roasting (typically operated above 600 °C) and emerging controlled-atmosphere roasting methodologies that achieve significant temperature reductions through thermodynamic optimization. While conventional high-temperature smelting demonstrates superior metal recovery rates, its long-term environmental sustainability remains questionable due to associated pollution risks. Modern roasting innovations present dual technical advancements: they not only enhance valuable metal recovery yields beyond conventional limits but also drastically reduce process energy requirements, aligning with industrial scalability criteria through improved thermodynamic efficiency and ecological compatibility.
(3) Hydrometallurgical processes encompass diverse leaching systems including acid-based, DES, and emerging specialized formulations. In acidic environments, inorganic acid leaching necessitates reducing agent addition to achieve effective metal dissolution. While inorganic acids offer economic advantages, their application raises concerns regarding environmental contamination and equipment degradation. Organic acid systems present an eco-friendly alternative with superior leaching kinetics, though their industrial implementation remains hindered by high operational costs. DES leaching systems represent a sustainable innovation, combining high extraction efficiency with solvent recyclability while minimizing toxic emissions. Despite requiring complex regeneration protocols, these systems exhibit significant research potential as future-oriented recycling solutions, particularly if process simplification can be realized for industrial scaling. Specialized leaching formulations have been developed to address the structural complexity of ternary lithium batteries, demonstrating efficient metal dissolution under tailored conditions using cost-effective reagents, thereby expanding technological options for sustainable battery recycling. Following solid–liquid conversion of target metals, selective recovery from pregnant leach solutions becomes critical. Current strategies primarily involve chemical precipitation and physical property-based separation techniques. Chemical precipitation maintains widespread adoption due to its process versatility, though its implementation often involves multi-step protocols and secondary waste generation. Physical separation methods, which exploit differences in solution chemistry (e.g., solubility product modulation), offer simpler, cleaner recovery pathways but exhibit narrower applicability compared to precipitation-based approaches.
(4) While existing policies and regulations support the advancement of the lithium-ion battery recycling sector, the industry currently lacks standardized protocols. This regulatory void has resulted in inconsistent recycling procedures and technical benchmarks, leaving numerous spent batteries unprocessed through safe or environmentally sustainable methods. To resolve these systemic challenges, future efforts should prioritize establishing uniform standards that ensure operational efficiency and environmental responsibility across the recycling value chain. Such measures will be critical to enabling sustainable industry expansion.

## 8. Conclusions

Driven by the global automotive electrification trend and the finite lifespan of LIBs, the core energy storage components in electric vehicles, the volume of discarded LIBs is poised to surge. Improper disposal of spent LIBs not only degrades environmental quality and endangers human health but also leads to significant waste of valuable metals such as lithium and cobalt. Therefore, efficient recycling of these metals is essential. Current mainstream recycling technologies include pyrometallurgy and hydrometallurgy. Hydrometallurgy can be further classified into acid leaching systems, DES leaching, and emerging leaching technologies, distinguished by their unique dissolution mechanisms.

Pyrometallurgy is suitable for treating complex systems containing lithium cobaltate or ternary cathode materials embedded in copper smelting slag, leveraging high-temperature phase changes to selectively liberate lithium while fixing transition metals. However, it faces challenges of high energy consumption and incomplete lithium extraction. Acid leaching systems are effective for highly selective metal separation: inorganic acid approaches excel in achieving high leaching rates at scale but require addressing equipment corrosion and impurity interference; organic acid systems, ideal for complex ternary materials, operate under mild conditions that suppress impurity dissolution—making them environmentally friendly—yet suffer from high acid consumption costs. DES leaching technology enables both single-metal extraction and synergistic recovery of multiple metals, avoiding the high pollution of traditional strong acids/bases. Nonetheless, its large-scale application is hindered by complex solvent regeneration and thermal decomposition of components at elevated temperatures. Emerging leaching systems, designed for high-stability ternary materials or specific cathode chemistries, improve recycling efficiency for dense, multi-metal systems but entail complex processes and substantial equipment investment.

While these methods exhibit niche advantages, they commonly struggle to balance energy use, cost, and environmental impact. To address the engineering needs for efficient recycling of waste ternary LIBs and cathode material regeneration, this study develops a closed-loop process integrating pre-treatment, low-temperature roasting, stepwise lithium–transition metal separation, and high-purity precursor synthesis.

In pre-treatment, PVDF binder is embrittled via 77 K cryogenic milling and detached through froth flotation—employing a 60 g/L slurry concentration and 250 g/t n-dodecane as a collector—to efficiently separate anode/cathode materials, yielding ≥99% pure anode fractions. A novel low-temperature roasting step at 300 °C uses Na_2_S_2_O_8_/Na_2_SO_4_ as a composite additive, converting lithium to water-soluble Li_2_SO_4_ while forming insoluble Ni/Co/Mn oxides. This reduces the roasting temperature by 400–500 °C compared to conventional processes, significantly cutting energy use and pollutant emissions. Water leaching of roasted products achieves a lithium extraction rate >98%, followed by stepwise alkali precipitation for impurity removal and lithium carbonate crystallization to produce ≥99.3% pure lithium salts.

For Ni/Co/Mn oxide slags, sulfuric acid leaching at 50 °C dissolves metals, after which impurity ions are removed and Ni^2+^/Co^2+^/Mn^2+^ concentrations are adjusted to a 1:1:1 ratio via co-precipitation, yielding spherical hydroxide precursor Ni_1/3_Co_1/3_Mn_1/3_(OH)_2_. Finally, the precursor is mixed with lithium salt, pre-calcined at 450 °C to remove volatiles, and sintered at 900 °C to regenerate LiNi_1/3_Co_1/3_Mn_1/3_O_2_—a layered cathode material with intact spherical morphology and electrochemical properties comparable to virgin materials.

This process surmounts traditional recycling bottlenecks, including high-temperature energy waste, compositional disorder, and performance degradation. By employing low-temperature energy reduction, precise compositional regulation, and high-value property retention, it delivers an engineering-feasible solution for the large-scale green recycling of waste ternary batteries.

Future research should prioritize two directions for LIB recycling. First, DES systems show significant potential for LIB recycling. Simplifying solvent recovery processes could enable DES-based methods to achieve cost-effective, efficient, and environmentally friendly metal recovery. Second, novel calcination technologies require more innovative designs to further reduce operating temperatures while maintaining energy-saving objectives.

## Data Availability

No new data were created or analyzed in this study.

## Abbreviations

The following abbreviations are used in this manuscript:

BDs	bean dregs
CA	citric acid
DES	deep eutectic solvents
DMSO	dimethyl sulfoxide
EG	ethylene glycol
EV	electric vehicle
FA	formic acid
ICP-OES	inductively coupled plasma optical emission spectrometry
Ksp	solubility product
LIBs	lithium-ion batteries
LCO	lithium cobalt oxide
LFP	lithium iron phosphate
BDs	bean dregs
EG	ethylene glycol
MCA	monochloroacetic acid
LNCM	ternary lithium battery cathode
TBAC	tetrabutylammonium chloride
LMFP	LiFe_x_Mn_1−x_PO_4_
LNCM	ternary lithium battery cathode
MCA	monochloroacetic acid
MIBC	methylisobutyl
NCA	nickel–cobalt–aluminum oxide
NMC	nickel–manganese–cobalt oxide
NMP	N-methyl-2-pyrrolidone
NTP	non-thermal plasma
OA	oxalic acid
PVDF	polyvinylidene fluoride
TBAC	tetrabutylammonium chloride
